# Supplementary material for: Genetic Association of the Functional WDR4 Gene in Male Fertility
Source: J Pers Med. 2021 Jul 30;11(8):760. doi: 10.3390/jpm11080760 (PMC8399419; doi:10.3390/jpm11080760)
Supplement: Supplementary file 1 [file jpm-11-00760-s001.zip › jpm-1245282-supplementary.pdf]

Article

Genetic association of the functional WDR-4 gene in male fertility

Yu-Jia Wang[1†], Eko Mugiyanto[2†], Yun-Ting Peng[1,5†], Wan-Chen Huang[3], Wan-Hsuan Chou[1,4], Chi-Chiu Lee[3], Yu-Shiuan Wang[2], Lalu Muhammad Irham[1,6], Dyah Aryani Perwitasari[6], Ming-I Hsu[7*], Wei-Chiao Chang[1,4,5,8*]

1 Department of Clinical Pharmacy, School of Pharmacy, College of Pharmacy, Taipei Medical University, Taipei 11031, Taiwan
2 PhD Program in Clinical Drug Development of Chinese Herbal Medicine, College of Pharmacy, Taipei Medical University, Taipei 11031, Taiwan
3 Institute of Cellular and Organismic Biology, Academia Sinica, Taipei 115, Taiwan
4 Master Program for Clinical Pharmacogenomics and Pharmacoproteomics, School of Pharmacy, Taipei Medical University, Taipei 11031, Taiwan
5 Department of Pharmacy, Wan Fang Hospital, Taipei Medical University, Taipei 11696, Taiwan
6 Faculty of Pharmacy, University of Ahmad Dahlan, Yogyakarta 55164, Indonesia
7 Department of Obstetrics and Gynaecology, Wan Fang Hospital, Taipei Medical University, Taipei 11696, Taiwan
8 Integrative Research Center for Critical Care, Wan Fang Hospital, Taipei Medical University, Taipei 11696, Taiwan
* Correspondence: Wei-Chiao Chang; email: wcc@tmu.edu.tw
Ming I Hsu; email: hsumingi@yahoo.com.tw

Supplementary Figure

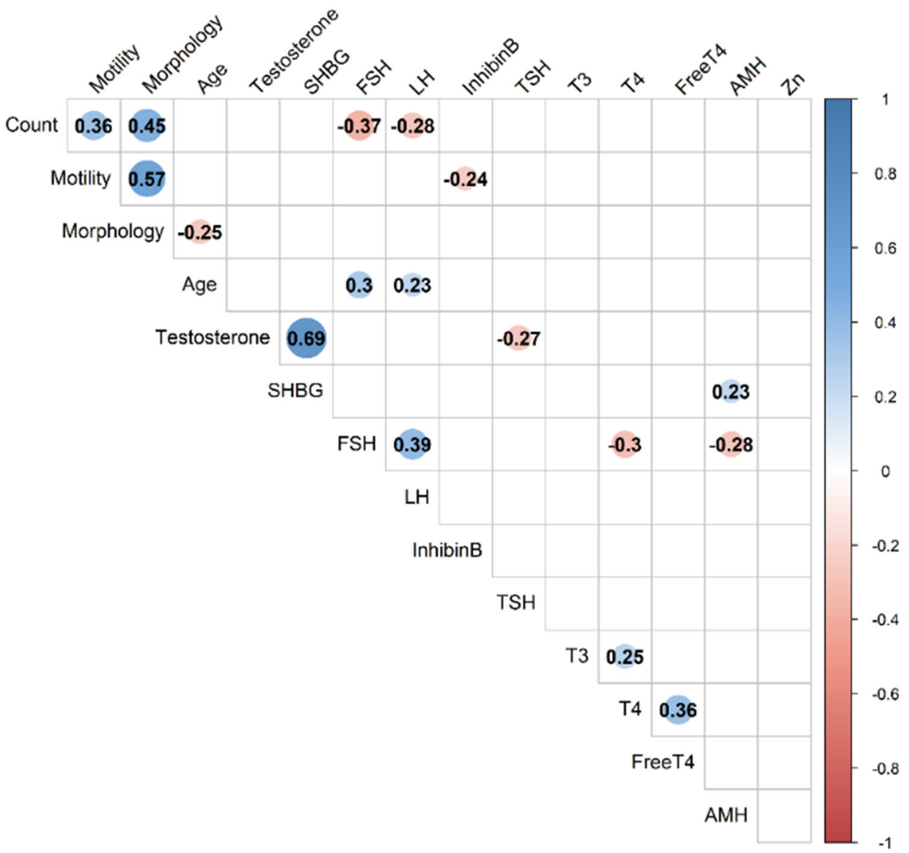

Figure S1. Spearman's rank correlation coefficient between age, serum hormones, and semen parameters

Supplementary Table

Table S1. Characteristics of tagging single-nucleotide polymorphisms (tSNPs) of the *WDR-4* gene

Gene	Location (GRCh37.p13)	SNP	Allele		Function	AFR Freq	AMR Freq	EUR Freq	ASN Freq	TWB Freq	Our study Freq
			Ref	Alt							
WDR4	chr21:44274642	rs2298666	G	A	Intron	0.03	0.06	0.05	0.17	0.15	0.15
	chr21:44275979	rs465663	T	C	Intron	0.24	0.32	0.32	0.32	0.29	0.26
	chr21:44293744	rs2248490	C	G	Missense	0.28	0.37	0.47	0.34	0.32	0.33
	chr21:44269355	rs3746939	A	C	3'-UTR	0.08	0.11	0.14	0.27	0.22	0.25

Freq, frequency of the alternate (Alt) allele. Ref, reference. AFR, African; AMR, American; ASN, Asian; EUR, European; TWB, Taiwan Biobank.

Table S2. *Cis*-expression quantitative trait loci (*cis*-eQTL) of rs465663

SNP	Gene	*p* value	NES	Tissue	Action
rs465663	*WDR4*	1.3x10^{-8}	0.37	Testis	TT<TC<CC
	WDR4	0.000043	0.12	Whole blood	TT<TC<CC
	WDR4	0.0001	0.10	Oesophagal mucosa	TT<TC<CC
	U2AF1	0.000031	-0.25	Artery tibial	TT>TC>CC
	U2AF1	0.000031	-0.25	Skin-not sun exposed (suprapubic)	TT>TC>CC
	U2AF1	0.000089	-0.19	Whole blood	TT>TC>CC
	U2AF1	0.00009	-0.23	Skin-sun exposed (lower leg)	TT>TC>CC
	NDUFV3	4.2x10^{-08}	-0.21	Cells: cultured fibroblasts	TT>CC>TC
	NDUFV3	3.6x10^{-06}	-0.16	Muscle-skeletal	TT>TC>CC
	NDUFV3	0.000034	-0.16	Esophagus-muscularis	TT>TC>CC
	LINC01668	4.9x10^{-07}	-0.29	Nerve-tibial	TT>TC>CC
	ERVH48-1	8.6x10^{-06}	0.19	Thyroid	TT<TC<CC
	CBS	2.8x10^{-06}	0.31	Cells: cultured fibroblasts	TT<TC<CC
	AP001628.7	8.0x10^{-07}	-0.29	Nerve-tibial	TT>TC>CC

SNP, single-nucleotide polymorphism; NES, normalized effect size. Data were obtained from the Genotype-Tissue Expression (GTEx) Portal on May 4, 2020.

Table S3. Associations between genetic variants of the *WDR4* gene and total sperm number

SNP	Genotype	No	Value		*p*-value		
			Mean	S.E.	Dominant	Recessive	Additive
rs2298666	GG	58	221.50	25.85	0.924	0.801	0.995
	GA	20	217.81	47.97			
	AA	2	254.00	76.00			
rs465663	TT	42	193.83	29.88	0.314	0.427	0.279
	TC	27	228.79	35.89			
	CC	10	290.92	74.94			
rs2248490	CC	38	213.36	34.27	0.734	0.549	0.974
	CG	33	242.80	35.85			
	GG	10	218.60	48.40			
rs3746939	AA	45	209.71	28.78	0.503	0.531	0.428
	AC	31	238.43	39.16			
	CC	5	295.60	80.12			

The *p*-value was adjusted for age, total testosterone, follicle-stimulating hormone, and luteinizing hormone. SNP, single-nucleotide polymorphism. S.E., standard error.

Table S4. Associations between genetic variants of the *WDR4* gene and sperm progressive motility

SNP	Genotype	No	Value		*p*-value		
			Mean	S.E.	Dominant	Recessive	Additive
rs2298666	GG	58	37.93	2.27	**0.014***	0.587	**0.021***
	GA	20	46.75	3.84			
	AA	2	51.50	1.50			
rs465663	TT	42	36.26	2.87	**0.025***	0.329	**0.041***
	TC	27	44.11	3.36			
	CC	10	48.90	0.85			
rs2248490	CC	38	35.53	3.12	**0.017***	0.617	0.050
	CG	33	44.36	2.78			
	GG	10	45.20	2.92			
rs3746939	AA	45	37.76	2.66	0.119	0.430	0.113
	AC	31	43.19	3.13			
	CC	5	45.60	4.48			

The *p*-value was adjusted for age, total testosterone, follicle-stimulating hormone, and luteinizing hormone. $*p<0.05$. SNP, single-nucleotide polymorphism. S.E., standard error.

Table S5. Associations between genetic variants of the *WDR4* gene and sperm morphology

SNP	Genotype	No	Value		*p*-value		
			Mean	S.E.	Dominant	Recessive	Additive
rs2298666	GG	58	10.48	0.61	0.799	0.251	0.900
	GA	20	9.55	0.84			
	AA	2	15.00	0			
rs465663	TT	42	10.31	0.79	0.855	0.961	0.915
	TC	27	9.96	0.73			
	CC	10	11.20	0.92			
rs2248490	CC	38	10.05	0.82	0.723	0.570	0.994
	CG	33	10.58	0.71			
	GG	10	10.30	1.16			
rs3746939	AA	45	10.49	0.73	0.624	0.436	0.922
	AC	31	9.77	0.68			
	CC	5	11.80	2.06			

The *p*-value was adjusted for age, total testosterone, follicle-stimulating hormone, and luteinizing hormone. SNP, single-nucleotide polymorphism. S.E., standard error.
